# Supplementary material for: The Effects of Aging on Rod Bipolar Cell Ribbon Synapses
Source: Cells. 2023 Sep 29;12(19):2385. doi: 10.3390/cells12192385 (PMC10572008; doi:10.3390/cells12192385)
Supplement: Supplementary file 1 [file cells-12-02385-s001.zip › cells-2593811-supplementary.pdf]

cells

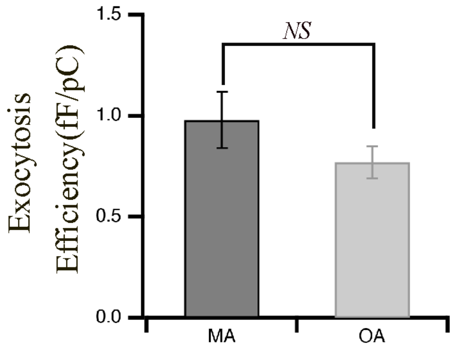

Figure S1. Retinal bipolar cells isolated from old-aged zebrafish exhibited no changes in their exocytosis efficiency relative to middle-aged fish. Exocytosis efficiency, obtained from the ratio of capacitance jump to Ca^{2+} current charge with pipe.

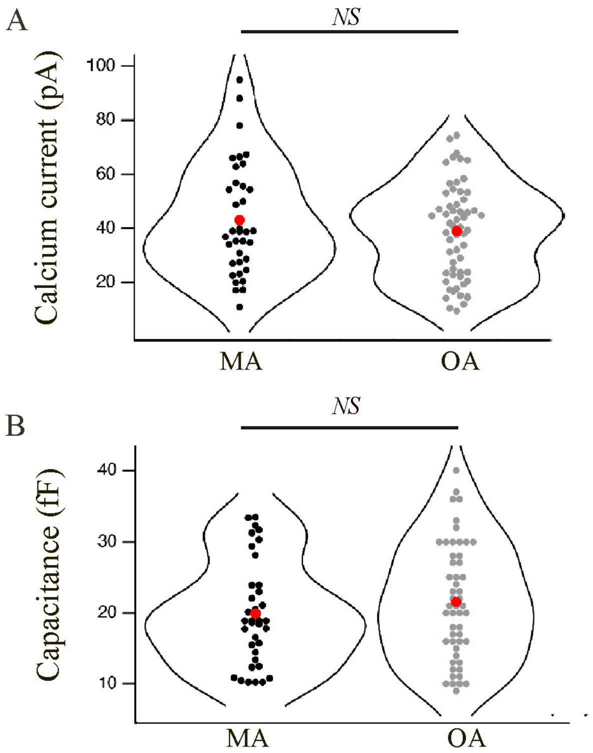

Figure S2. Retinal bipolar cells isolated from old-aged zebrafish exhibited no changes in their brief release properties relative to middle-aged fish. A. Ca^{2+} current (I) recorded from the synaptic terminal of a bipolar neuron isolated from middle-aged (MA, black-filled circle) and older-aged (OA, gray-filled circle) zebrafish in response to a voltage-clamp pulse (V) from −60 mV to -15 mV for 10 ms. Note: Each point represents individual calcium currents obtained from the same cell multiple times or different cells. The average calcium current is represented in red circles. B. Capacitance in response to a voltage-clamp pulse (V) from −60 mV to -15 mV for 10 ms that was obtained from bipolar neurons of MA (A, black-filled circle) and OA (B, gray-filled circle) zebrafish. Note: Each point represents individual capacitance measurements obtained from the same cell multiple times or different cells. The average capacitance measurements are represented in red circles. N= 20 MA, seven animals: N=12 OA bpcs; nine animals.
